# Management of superficial venous thrombosis based on individual risk profiles: protocol for the development and validation of three prognostic prediction models in large primary care cohorts

**DOI:** 10.1186/s41512-021-00104-8

**Published:** 2021-08-18

**Authors:** F. S. van Royen, M. van Smeden, K. G. M. Moons, F. H. Rutten, G. J. Geersing

**Affiliations:** 1 Dept. General Practice, Julius Center for Health Sciences and Primary Care, University Medical Center Utrecht, Utrecht University, Utrecht, Netherlands; 2 Dept. Epidemiology, Julius Center for Health Sciences and Primary Care, University Medical Center Utrecht, Utrecht University, Utrecht, Netherlands

**Keywords:** Superficial venous thrombosis, Thrombophlebitis, Primary care, Prediction, Prognosis

## Abstract

**Background:**

Superficial venous thrombosis (SVT) is considered a benign thrombotic condition in most patients. However, it also can cause serious complications, such as clot progression to deep venous thrombosis (DVT) and pulmonary embolism (PE). Although most SVT patients are encountered in primary healthcare, studies on SVT nearly all were focused on patients seen in the hospital setting. This paper describes the protocol of the development and external validation of three prognostic prediction models for relevant clinical outcomes in SVT patients seen in primary care: (i) prolonged (painful) symptoms within 14 days since SVT diagnosis, (ii) for clot progression to DVT or PE within 45 days and (iii) for clot recurrence within 12 months.

**Methods:**

Data will be used from four primary care routine healthcare registries from both the Netherlands and the UK; one UK registry will be used for the development of the prediction models and the remaining three will be used as external validation cohorts. The study population will consist of patients ≥18 years with a diagnosis of SVT. Selection of SVT cases will be based on a combination of ICPC/READ/Snowmed coding and free text clinical symptoms. Predictors considered are sex, age, body mass index, clinical SVT characteristics, and co-morbidities including (history of any) cardiovascular disease, diabetes, autoimmune disease, malignancy, thrombophilia, pregnancy or puerperium and presence of varicose veins. The prediction models will be developed using multivariable logistic regression analysis techniques for models i and ii, and for model iii, a Cox proportional hazards model will be used. They will be validated by internal-external cross-validation as well as external validation.

**Discussion:**

There are currently no prediction models available for predicting the risk of serious complications for SVT patients presenting in primary care settings. We aim to develop and validate new prediction models that should help identify patients at highest risk for complications and to support clinical decision making for this understudied thrombo-embolic disorder. Challenges that we anticipate to encounter are mostly related to performing research in large, routine healthcare databases, such as patient selection, endpoint classification, data harmonisation, missing data and avoiding (predictor) measurement heterogeneity.

## Background

Superficial venous thrombosis (SVT) is an inflammatory process coincided with thrombus formation in the superficial venous system, most commonly of the legs. The clinical manifestation consists of a red, swollen and painful venous cord [1]. SVT is one of several conditions together called venous thromboembolism (VTE), which also includes the diagnoses deep venous thrombosis (DVT) and pulmonary embolism (PE). SVT is generally less well-known and studied than DVT and PE, likely because of its assumed benign character by many physicians. Consequently, many unanswered questions remain on the outcome and management of SVT, notably for those patients managed in primary care.

Research on SVT is relatively scarce and if performed, studies usually involve patients referred to hospital settings. For several reasons the results from research on SVT performed in hospital care settings are not easily generalizable to primary care settings. A study in referred SVT patients reported an incidence rate of 0.64 per 1000 person-years [2], while a study performed in primary care using routine clinical data estimated a much higher incidence rate of 1.31 per 1000 person-years follow-up, a number similar to the incidence rates of DVT and PE [3, 4]. This difference in incidence rates can be explained, at least in part, by selective referral of patients with the most severe SVT signs and symptoms, and possibly a VTE history. In general, prognostic prediction of more severe SVT patients seen in hospital settings is not generalizable to SVT patients seen in primary care.

While SVT in primary care is often considered a benign condition that resolves naturally without the administration of any treatment, a subset of patients indeed does develop severe complications, such as clot progression to deep venous thrombosis (DVT) and pulmonary embolism (PE). Since these conditions need anticoagulant intervention in order to prevent further complications like right ventricular dysfunction, shock and ultimately death related to PE, identifying the patients most at risk for severe complications is pivotal. Furthermore, some SVT patients suffer from prolonged painful symptoms, SVT extension or SVT recurrence [5]. Data on hospital referred SVT patients suggest that between 6 and 53% of SVT patients suffer from concomitant DVT or PE and that approximately 3–15% of initially isolated SVT cases eventually develop VTE clot progression, mostly within 1 month [2, 3, 6–9].

Preventive anticoagulant treatment has been proposed in patients at high risk of developing thrombotic complications [10, 11]. It is, however, currently unclear what defines a high-risk patient and how risk should be inferred or calculated. While some patient characteristics have been reported in literature to be more prevalent in patients with clot progression (such as active malignancy and absence of varicose veins) [3, 5, 12], it remains uncertain which patients are more likely to suffer from SVT complications and would benefit from early intervention in order to prevent thrombotic complications. Since the treatment can cause anticoagulant induced bleeding, selecting only the patients at highest risk thrombosis risk is important [10, 13].

In this article, we present the protocol for the development and external validation of three prognostic prediction models for three different clinical outcomes of SVT in primary care: (i) prolonged (painful) symptoms within 14 days since SVT diagnosis, (ii) for clot progression within 45 days and (iii) for clot recurrence within 12 months. These models will be developed with the ultimate aim to improve risk estimation in SVT patients presenting in primary care, to improve tailored treatment decision making and to better prevent severe SVT complications.

## Methods

### Study design and setting

This study involves an analysis of routinely collected clinical data from four primary care registries from the Netherlands (Intercity Consortium and Pharmo General Practitioner Database) and the UK (SAIL Databank, CPRD Gold and Aurum). Details of the four registries that will be used are shown in Table 1 [14–17]. Together, these databases entail several millions of primary care patients.

**Table 1:** Characteristics of primary care registry databases that will be approached for this study

Name of database	Country	Number of patients	Data coding	Free text available	Linkage to hospital data available	Estimated number of SVT cases^a^	Intended use of database: development/validation
Intercity Consortium [14]	The Netherlands	1M	ICPC codes	Yes	No	13,100	Validation
Pharmo General Practitioner Database [15]	The Netherlands	2.5M	ICPC codes	Yes	Yes	32,750	Validation
SAIL databank [16]	Wales	3.5M	Read codes	No	Yes	45,850	Validation
CPRD Gold and Aurum [17]	England	50M	Read and Snowmed codes	No	Yes	655,000	Development

^a^Based on an incidence rate of 1.31 per 1000 person-years and the assumption of 10 years follow-up [3]

*ICPC* International Classification of Primary Care

### Study population

The study population is defined by patients with a diagnosis of SVT in primary care based on clinical symptoms (red, swollen and tender venous cord), with or without confirmation by ultrasonography. Patients are eligible for inclusion if 18 years or older with a diagnosis code in the electronic medical record corresponding to SVT, and/or with SVT described as the main diagnosis in free text and clinical symptoms clearly fitting this diagnosis. The exclusion criteria are as follows: SVT considered as a differential diagnosis but not registered as the main diagnosis, SVT mentioned as part of a patient’s medical history and not part of a (recent) consultation or a clinical description that does not fit SVT diagnosis. Data that were collected between 2000 and 2020 will be used.

All general practitioners (GPs) contributing to the Dutch and UK primary care registries use disease coding to link clinical findings from signs and symptoms to the electronic medical record. Selection of patients will be based on these clinical disease coding systems: International Classification of Primary Care (ICPC) coding in Dutch databases and READ/Snowmed coding in UK-based databases. The UK READ/Snowmed coding is more detailed than the Dutch ICPC system; therefore, additionally, all patient contacts in the Dutch databases will be assessed for SVT and a variety of synonyms by automated free text searching. Additional linkage to clinical hospital or pharmacy data for confirmation of SVT diagnosis, predictor data collection or endpoint classification is preferred but might be limited due to database structures.

We anticipate heterogeneity in the patients selected, predictor data and endpoint classifications due to the differences in data granularity of reporting systems as well as ethical and privacy related constraints that differ between the databases. These differences in data structure and available patient information between databases will be thoroughly described in our study and the degree of heterogeneity will be investigated (Table 1).

### Data collection

Prediction baseline is determined by the moment of SVT diagnosis by the GP. The following patient characteristics will be collected: patient’s age in years, sex, body mass index (BMI), current or past smoking status and medical history of any venous thrombotic event (DVT/PE/SVT). Data on patients’ co-morbidities will be extracted by screening for disease coding and free text including (history of any) cardiovascular disease, diabetes, autoimmune disease, malignancy, thrombophilia, pregnancy or puerperium at moment of diagnosis and presence of varicose veins. Active medication prescribed within the last 3 months before diagnosis will also be extracted to assess as possible predictors. From free text consultations (or alternatively using disease coding if appropriate) up to 12 months post SVT diagnosis, information on length of the inflamed vein (in cm), location (distance to the saphenofemoral junction (SFJ)), symptoms, treatment of SVT (conservative, stockings, analgesics, anticoagulation), progression to DVT/PE within 45 days and recurrence of SVT within 12 months will be collected.

### Study endpoints

**Fig. 1:**
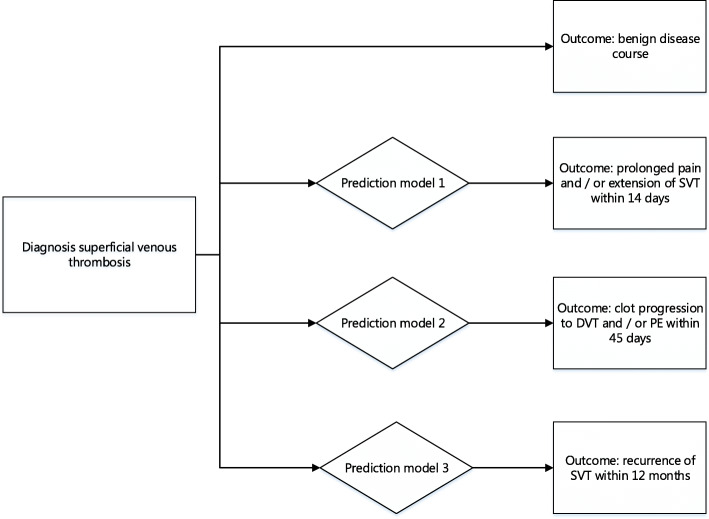
Flow diagram of possible disease trajectories of superficial venous thrombosis patients

This study will focus on the development and validation of three prediction models for three separate pre-defined post-SVT diagnostic outcomes. The endpoint of the first model is prolonged (painful) symptoms defined as any re-consultation within 14 days after SVT diagnosis with symptoms of pain and/or any extension of the SVT clot to the saphenofemoral junction (SFJ); for the second model, the endpoint is clot progression to DVT and/or PE within 45 days after SVT diagnosis; and for the third model, the endpoint is recurrent SVT within 12 months (Fig. 1).

Prolonged (painful) symptoms will be assumed present if the patient had a re-consultation for SVT and free text stating extension or pain within 14 days and new medication was prescribed (e.g. anticoagulants for extension of SVT or analgesics for painful symptoms). Clot progression within 45 days is deemed to be present if a hospital referral was made and the radiological report was positive for VTE, and/or if the hospital discharge letter stated diagnosis of VTE, and if anticoagulant medication in dosages typically used for DVT or PE was prescribed. Recurrent SVT is defined as a second episode of SVT within 12 months, mentioned in free text or as new ICPC/READ/Snowmed code. Endpoints will be measured using disease coding by the GPs in the electronic medical records. For endpoint assessment in the Dutch databases, automated free text searching for synonyms of painful symptoms, clot progression and SVT recurrence will be used additionally.

### Statistical analysis

The prognostic prediction models will be developed using multivariable logistic regression analyses techniques in the first and second model. For the third model with the outcome recurrence of SVT within 12 months, Cox proportional hazards modelling techniques will be used. Primary predictor selection will be based on clinical expertise. Baseline patient characteristics, age, co-morbidities, VTE history including history of SVT and SVT characteristics such as extension and location will be considered as predictors. In order to be able to select predictors during the modelling process, we will apply an elastic net penalty based on 10-fold cross-validation, minimising the deviance. Age will be evaluated as a continuous variable and non-linearity will be accounted for using restricted cubic splines with 4 knots. To explore the influence of received anticoagulants treatment on the outcome, we will consider the following techniques: (i) develop a model in non-anticoagulated patients first and test the performance in patients having received anticoagulation and (ii) using a more advanced statistical approach based on marginal structural models as described by Sperrin et al. [18]. The largest database, the UK-based CPRD databank, will be used for development of the models and for geographical and temporal internal-external cross-validation, i.e. by iteratively excluding different geographic regions in the derivation process and validate the model in the region initially left out. Next, to be able to understand heterogeneity between the databases and to test the transportability of the model to other populations and contexts, the models will be externally validated in the other three datasets (Table 1). For validation of the models, calibration and discrimination will be evaluated: observed and predicted events will be quantified in calibration plots, and for discrimination, decision curve and c-statistic will be quantified. For decision curves, the full range of estimated risk will be considered in the absence of a clear treatment guideline for SVT. In reporting the prediction models, we will adhere to the TRIPOD statement [19].

### Missing data

Due to the use of routine clinical data, missing data will likely be an issue. Patterns and possible causes for missing data will be investigated after the data are collected. Based on these findings, a decision will be made on how to handle the missing values in the analyses. We anticipate we will be using multiple imputation for predictors with missing data, assuming the missingness at random assumption (MAR) is plausible and the missingness of the candidate predictor is less than 50%. Otherwise, the predictor will no longer be considered a candidate.

### Sample size calculation

In a recent study in primary care, it was shown that approximately 4% of SVT patients suffer from clot progression, i.e. the event fraction for the second proposed model for clot progression to DVT or PE [3]. Using the sample size calculation for clinical prediction models proposed by Riley et al. [20], a sample size of at least 4420 SVT patients is required for allowing 25 predictors, including interaction terms, for model development with 177 events, an events per predictor (EPP) number of 7.07 and using a *R*^2^_cs_ of 0.0495. This *R*^2^_cs_ is an estimation in absence of a known value, if varying the *R*^2^_cs_ from 0.0595 to 0.0395 this results in a (minimal) sample size ranging from 3656 to 5571 SVT patients. For the other two prediction models, the event fractions are yet unknown hampering our possibility to reliably estimate a minimum sample size at this point. Nevertheless, we expect the event fraction for both models to be more or less similar as to the event fraction for model 2, with the exception for model 1 (prolonged SVT symptoms) for which a higher event fraction is likely to be encountered. Based on Table 1, we expect that this sample size will be easily achieved using our routine healthcare databases and we therefore conservatively aim to include at least 10,000 SVT patients for the studies described in this protocol.

## Discussion

In this paper, we describe the protocol for the development and validation of three prognostic models for patients suffering from superficial venous thrombosis using multiple large international primary care cohort. The overarching aim is to evaluate the individual SVT patient’s prognosis or disease trajectory. By developing and validating three separate risk prediction models for possible adverse outcomes in SVT patients (prolonged symptoms, clot progression to DVT and/or PE and SVT recurrence), this study will inform physicians on possible disease trajectories in patients with SVT and thus contribute to evidence-based decision making in the management of SVT in primary care.

Prediction models and risk scores have long been used in VTE management: the Wells rule for DVT and pulmonary embolism, as well as the YEARS algorithm for suspected pulmonary embolism are well-known and widely integrated examples [21–26]. These models have been validated and improved extensively and have proven their use in many clinically relevant subgroups or domains. In this respect, SVT has remained behind. Pomero and colleagues tried to develop a risk score, the ICARO score, for the concomitant presence of DVT and SVT in patients [27]. They identified age >50 years, presence of cancer, oedema of the limb, rope-like sign (the feeling of a swollen venous cord) and an idiopathic event (no apparent cause for SVT) as predictors for concomitant DVT. The score classifies patients in three risk categories: low risk, intermediate risk and high risk. Used in clinical practice, the score was meant to prevent unnecessary diagnostic imaging. However, upon external validation, it failed to demonstrate sufficient predictive performance, exemplified by very poor discriminative properties with an area under the ROC curve reported of 0.39 (95% CI 0.27–0.50), a sensitivity of 36.0%, a specificity of 40.2%, a positive predictive value of 13.4% and a negative predictive value of 70.9%. These numbers could possibly be explained by the differences in study design of the development study and the validation study and the low number of events in the validation study [28]. Furthermore, the model was developed in a relatively small dataset of 494 SVT patients and the study population was selected based on echo-colour Doppler or ultrasonography confirmed SVT and did not reflect the real clinical practice population wherein certainly not all patients would undergo these additional tests. Thus, we hypothesise that using routine clinical care data in combination with a large SVT population will likely yield more stable predictions.

There are currently no prognostic prediction models for the risk stratification and management of SVT in clinical use, making its clinical management ambiguous, uncertain and in the end suboptimal. This puts patients at risk of thrombo-embolic complications if patients are wrongly left untreated based for instance upon a presumed low risk of clot progression. Nevertheless, it also puts patients unnecessarily at risk of bleeding complications if a low-risk SVT patient is given antithrombotic treatment. With this project, we hope to provide valuable novel evidence to optimise the treatment of this understudied thrombo-embolic disorder.

### Challenges and limitations

There are some challenges we anticipate encountering while conducting this research, mostly relating to performing research in large, routine healthcare databases. First, the selection of patients in each database based on ICPC/READ or Snowmed coding alone might not be sufficient as it is imaginable that general practitioners will in some cases use a different code, for instance the general code for VTE, or no code at all. Free text screening is necessary, notably text mining procedures like natural language processing. Similarly, this is the case for endpoint classification as adverse outcome might be measured (only) by clinical symptom reporting, referral notes and/or the initiation of any treatment, notably for the outcome of prolonged SVT symptoms.

A limitation of this research is that we will only be able to include patients that have consulted their general practitioner. Patients that have directly gone to the hospital emergency department or to primary care out-of-hours services and likely suffering from more severe symptoms of SVT will be missed, albeit we expect that this will be only the case for the minority of patients in healthcare systems that are similar to the Dutch and UK systems. Ensuring overall predictor quality will be another challenge: missing values, different timing of predictors and measurement errors due to inter- and intra-observer variability between primary care physicians. However, by introducing three large datasets for external validation, we hope that it is possible to understand (and where possible adjust for) this heterogeneity in predictor assessment between the databases and conclude on the translatability of the models to different clinical cohorts. Nevertheless, with these limitations in mind, performing this research using routine healthcare data remains an important addition to the sparse literature of managing SVT in primary care.

## Conclusion

Superficial venous thrombosis is mainly encountered in primary care, and despite being associated with a benign disease course in most patients, complications in a small number of patients could have serious consequences. Knowledge on patients at risk of a complicated outcome is lacking, and at this point, treatment of SVT remains a shot in the dark. A stratified approach, thus separating those at higher risk of complications from the low-risk population, could provide valuable information that may inform physicians in order to improve clinical decision making in (primary care) SVT patients.

## Data Availability

The data that will be used for this study are available from the routine primary care registries CPRD Gold and Aurum databank (UK-based), Intercity Consortium (based in the Netherlands), Pharmo General Practitioner Database (based in the Netherlands) and SAIL databank (UK-based). Restrictions apply to the availability of these data, which will be used under licence for the current study, and so are not publicly available. Data will however be available from the authors upon reasonable request and with permission of the individual registries.

## Abbreviations

BMI: Body mass index
DVT: Deep venous thrombosis
GP: General practitioner
ICPC: International classification of primary care
PE: Pulmonary embolism
SFJ: Saphenofemoral junction
SVT: Superficial venous thrombosis
VTE: Venous thromboembolism
